# Novel insights into the role of metabolic disorder in osteoarthritis

**DOI:** 10.3389/fendo.2024.1488481

**Published:** 2024-12-18

**Authors:** Congcong Yu, Siyu Zhao, Songkai Yue, Xiaoyang Chen, Yonghui Dong

**Affiliations:** Department of Orthopedics, Henan Provincial People’s Hospital, Zhengzhou University People’s Hospital, Zhengzhou, China

**Keywords:** osteoarthritis, metabolism, glycolysis, amino acid, fatty acid

## Abstract

Osteoarthritis (OA) is a prevalent condition that affects individuals worldwide and is one of the leading causes of disability. Nevertheless, the underlying pathological mechanisms of OA remain inadequately understood. Current treatments for OA include non-drug therapies, pharmacological interventions, and surgical procedures. These treatments are mainly focused on alleviating clinical manifestations and improving patients’ quality of life, but are not effective in limiting the progression of OA. The detailed understanding of the pathogenesis of OA is extremely significant for the development of OA treatment. Metabolic syndrome has become a great challenge for medicine and public health, In recent years, several studies have demonstrated that the metabolic syndrome and its individual components play a crucial role in OA. Consequently, this review summarizes the mechanisms and research progress on how metabolic syndrome and its components affect OA. The aim is to gain a deeper understanding of the pathogenesis of OA and explore effective treatment strategies.

## 1 Introduction

Osteoarthritis(OA) is a prevalent degenerative disease that can affect nearly any joint throughout the human body, with the knee and hip joints are the areas most frequently affected. Epidemiological studies have found that more than 300 million people worldwide suffer from OA, which reduces the quality of life of patients and brings economic burden to individuals and society (1, 2). OA will cause a series of symptoms including joint pain, stiffness, dysfunction and limited mobility. Risk factors for OA include age, genetics, joint structure, obesity, trauma, and physical activity, all of which can affect the progression of OA (3), in addition to the fact that some studies have found that hypertension, cardiovascular disease, and diabetes increase the risk of developing OA and have a poorer prognosis (4–6). At present, the treatment plans for OA encompass exercise, weight management, medication therapy, surgical treatment, etc. The commonly used medications include non-steroidal anti-inflammatory drugs, acetaminophen, intra-articular injections of corticosteroids, etc (7), which cannot reverse the progression of OA. Total joint replacement surgery is not appropriate for young patients due to its limited service life (7). Consequently, it is essential to gain a deeper understanding of the pathogenesis of OA in order to develop more precise and effective treatments for patients with OA and to reduce the overall burden of OA on the healthcare system and society.

The pathogenesis of OA is complex, and although several researches have been studied on OA, its pathogenesis is still poorly understood, resulting in poor treatment outcomes. Cartilage degeneration is an important pathologic feature of OA (8), and in recent years with more research it has been found that all joint tissues are affected by OA, including bone, synovium, menisci, tendons, ligaments, and the infrapatellar fat pad (IFP), of which inflammatory and fibrotic changes of the IFP promote the progression of OA, leading to its being stiffer in comparison to other parts of the adipose tissue (9), and its volume and morphology correlate with OA (10–12). The progression of OA involves several factors, including inflammatory, mechanical, and metabolic processes (13). It is widely recognized that persistent low-grade inflammation plays a key role in OA (14), and multiple pro-inflammatory factors such as IL-1, IL-6 and TNF-α are elevated in OA (15). These pro-inflammatory factors are associated with joint fibrosis, and most OA patients have synovial fibrosis (16), which is associated with overexpression of TGF-β. Over-expression of TGF-β can also regulate the progression of OA through Smad2/3 (17). Mechanical stimulation is important for chondrocytes (18), and it has been found that chondrocytes regulate the progression of OA through mitochondrial perception of environmental mechanical signals (19), Various factors leading to alterations in the biomechanical environment of the joint can exacerbate the progression of OA (20). Furthermore, some studies have shown that metabolic reprogramming is essential for the onset of inflammation (21, 22). In healthy joints, chondrocytes maintain a state of metabolic homeostasis (23). In OA, there is a transition of energy metabolism from a resting state to a more active metabolic condition (24). The evidence suggests that metabolic flexibility is significantly impaired in OA (25).

A cross-sectional study enrolling 5764 participants found that components of the metabolic syndrome such as hypertension, dyslipidaemia, and central obesity were associated with the prevalence of knee OA, and that the prevalence of knee OA increased with the accumulation of metabolic syndrome components (26). Meanwhile an 8-year longitudinal study found a positive correlation between obesity and the prevalence of OA (27). Similarly, in another longitudinal study, metabolic syndrome was found to be associated with the progression of knee OA (28). Interestingly a study found an association between metabolic syndrome and hip OA in women but not in men, possibly due to the poor enrollment of participants (29). Alterations in the metabolism of chondrocytes are pivotal to the development of OA, encompassing disorders related to glycolysis, amino acids(AAs), lipids, and trace elements (8). There is a strong correlation between metabolic syndrome and its various components with OA. This review emphasizes the metabolic alterations and their underlying mechanisms in OA, aiming to offer new strategies for the diagnosis and treatment of the condition.

## 2 Methods

Firstly, we conducted a systematic review of the correlation between OA and metabolic disorders using PubMed to search the relevant literature until October 2024. The keywords included “‘metabolic syndrome’, ‘osteoarthritis’, ‘glycolysis’, ‘fatty acid’, ‘amino acid’, and ‘glycolysis’. ‘, ‘amino acid’. Based on the relevance of titles and abstracts, our inclusion criteria was to select only articles written in English, including reviews, *in vitro* and animal model studies, investigative studies, retrospective studies, and prospective studies, and exclusion criteria was to select articles written in languages other than English, including brief reports, commentaries, poster presentations and articles that did not clearly define. Finally, based on the literature that met the inclusion criteria, we summarised and discussed the association and mechanisms between metabolic disorders and OA.

## 3 Clinical correlation between metabolic syndrome and OA

**Figure 1:**
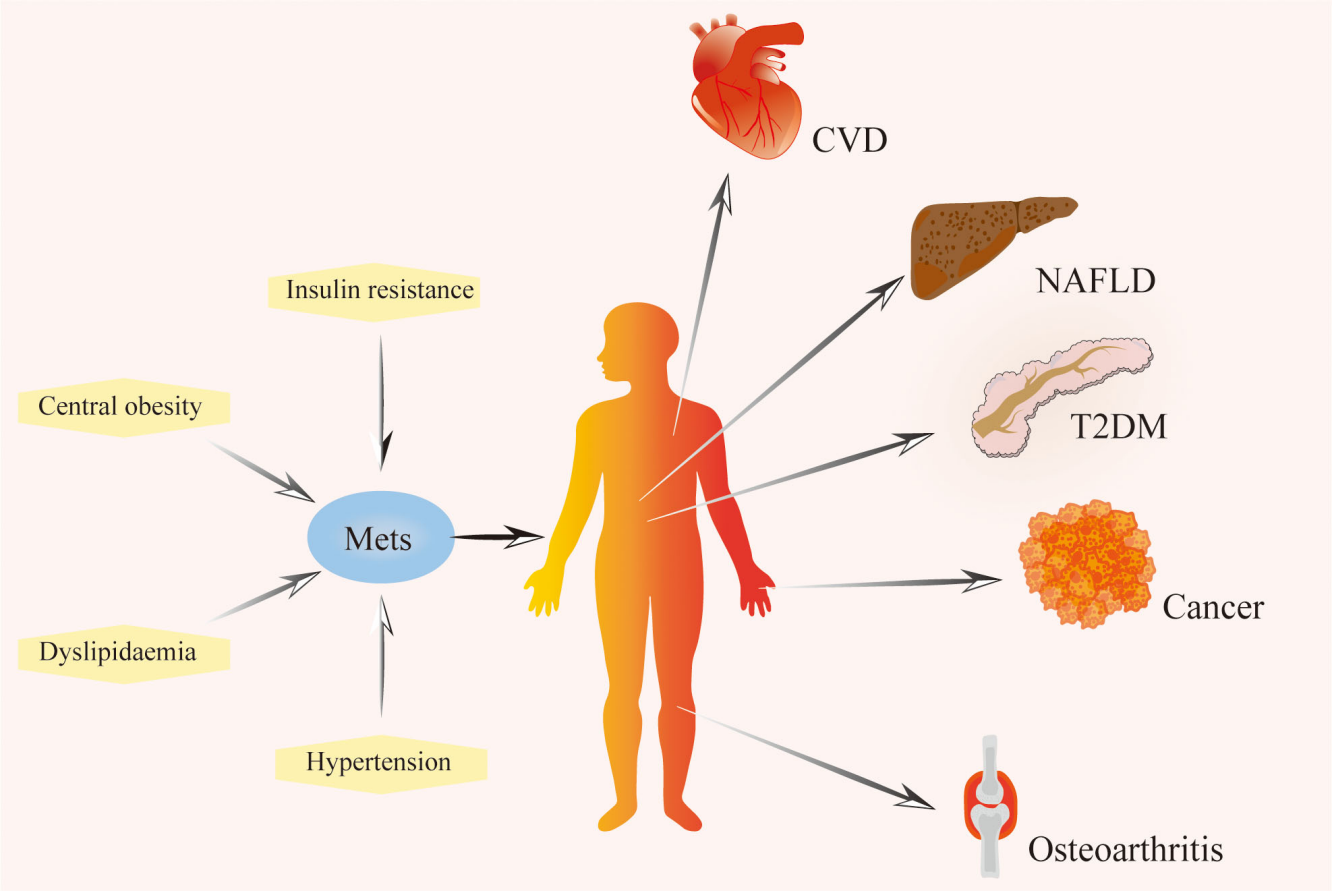
The composition of metabolic syndrome and related diseases. Metabolic syndrome is characterized by a series of metabolic disorders, including insulin resistance, dyslipidemia, hypertension, and central obesity. It is a risk factor for cardiovascular disease, non-alcoholic fatty liver disease, type 2 diabetes mellitus, cancer, osteoarthritis, and other diseases. Mets, Metabolic Syndrome; T2DM, Type 2 Diabetes Mellitus; NAFLD, Non-alcohol Fatty Liver Disease; CVD, Cardiovascular Disease.

Metabolic syndrome directly affects health, characterized by insulin resistance, atherogenic dyslipidemia, central obesity, and hypertension (30), and increased risk of Type 2 diabetes and cardiovascular disease (31–33), As a result, metabolic syndrome is recognized as a critical public health issue on a global scale (34). **Table 1** summarizes the diagnostic criteria for metabolic syndrome in different organizations. Metabolic diseases are increasingly prevalent and may impact multiple organs of the body (**Figure 1**). Through comprehensive investigation into OA and metabolic syndrome, new findings have recently surfaced that connect metabolic syndrome and its various components to the advancement of OA and its associated pain (35, 36). Metabolites can be used as biomarkers, alterations in body metabolites are crucial in the pathological mechanism of OA (37).

**Table 1:** Diagnostic criteria for metabolic syndrome.

Risk Factors	WHO(1998)	EGIR(1999)	IDF(2005)	NCEP-ATP III(2005)
Blood pressure	≥ 140/90 mmHg	≥ 140/90 mmHg	≥ 130 /≥ 85 mmHg	≥ 130 /≥ 85 mmHg
Fasting glucose	–	>6.1mmol/L	≥5.6mmol/L	≥5.6mmol/L
Central obesity	BMI> 30 kg/m^2^ or Men WHR>0.9 Women WHR>0.85	Waist circumference Men ≥94cm Women ≥80cm	Waist circumference Men ≥90cm Women ≥80cm	Waist circumference Men ≥102cm Women ≥88cm
HDL-C	Men <0.9 mmol/L Women <1.0 mmol/L	< 1.0 mmol/L	Men <0.9 mmol/L Women <1.3 mmol/L	Men < 1.03 mmol/L Women < 1.3 mmol/L
Triglycerides	≥ 1.7 mmol/L	>2.0 mmol/ L	≥ 1.7 mmol/L	≥1.7 mmol/L
Microalbuminuria	AER ≥ 20 µg/min or ACR≥30 mg/g	–	–	–

BMI, body mass index; WHR, waist hip ratio; HDL-C, high-density lipoprotein cholesterol; ACR, Albumin-to-creatinine ratio; AER, albumin excretion rate.

Obesity is a major component of the metabolic syndrome and an important risk factor for the progression of OA, but it is unclear whether the increased mechanical loading caused by obesity is resulting in the progression of OA or some other aspect of the metabolic syndrome (38). A Population-Based Cohort Study demonstrates that obesity increased the progression of hand OA and that the increased risk in women was mostly for hand OA (39), suggesting a systemic factor between obesity and OA. Obesity induces infiltration of synovial pro-inflammatory macrophages before the cartilage degeneration and promotes OA (40). Additionally, hyperglycemia plays a critical role in the pathophysiology of OA by facilitating the accumulation of advanced glycation end products within fibroblast-like synoviocytes. This accumulation leads to an increased release of inflammatory mediators that trigger chondrocyte degradation and advance OA progression (41). The findings of a meta-analysis showed that hypertension and metabolic syndrome were positively associated with OA, and dyslipidemia was not associated with OA (6), but the results of studies showed that disorders of lipid metabolism are likely to be involved in the pathogenesis of OA (42), so more research is needed to clarify the relationship between disorders of lipid metabolism and OA. Several clinical studies have shown that Metabolic Syndrome and its components are associated with the mechanisms of OA, but the analysis generally did not include adjustment for BMI, and after BMI adjustment, metabolic Syndrome and its components were not associated with OA (43), which requires further research.

## 4 Trace element and cartilage metabolism

**Figure 2:**
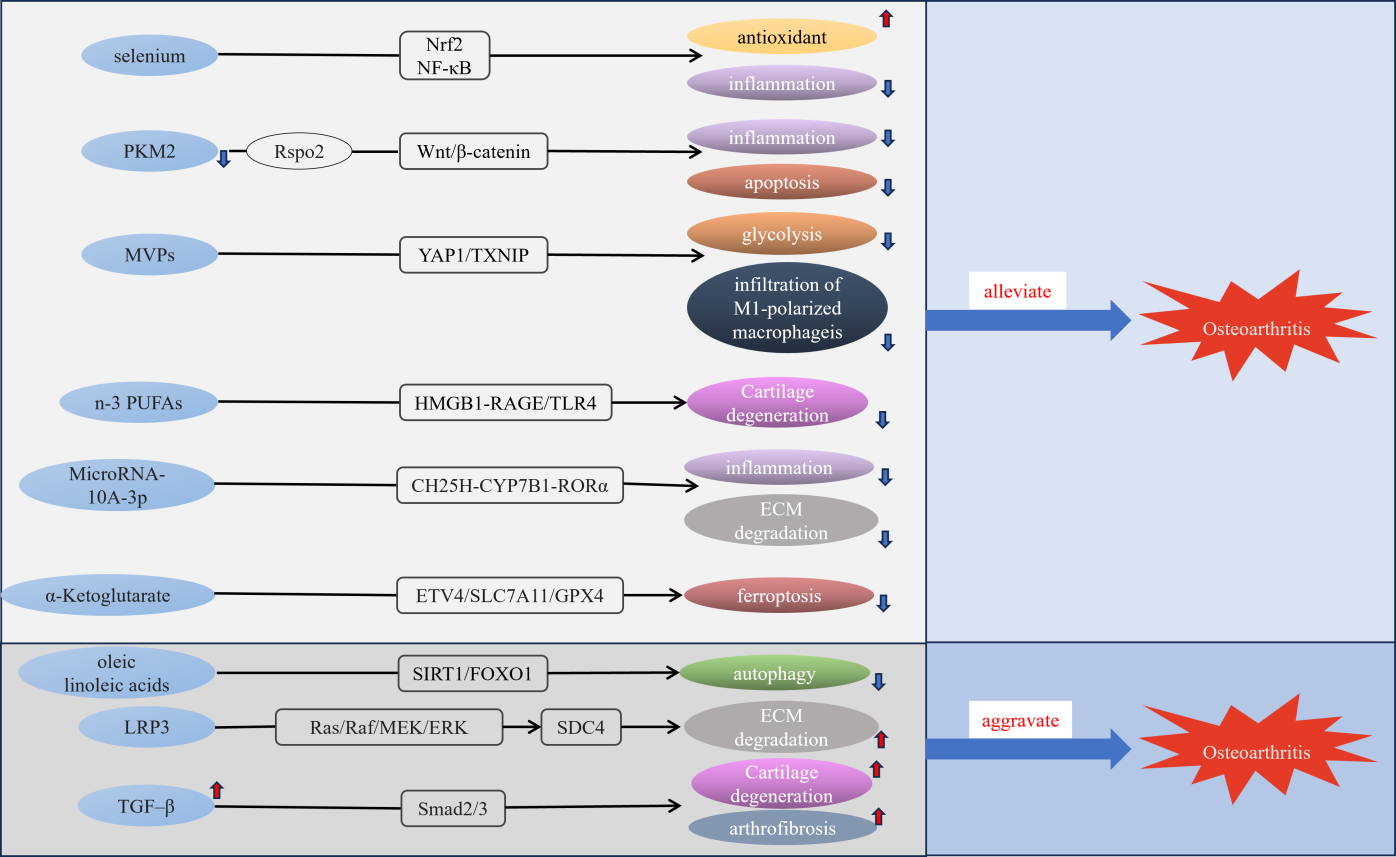
Molecular mechanisms of some metabolic disorders and OA. MVPs, M1 macrophage membrane-camouflaged Verteporfin (Vt)-loaded PLGA nanoparticles; n-3 PUFAs, n-3 polyunsaturated fatty acid; LRP3, low-density lipoprotein receptor-related protein 3.

Trace elements are essential components in various metabolic and regulatory processes within living organisms, and disorders of trace element metabolism could be linked to the development of various diseases, including cancer, gastrointestinal disorders, and cardiovascular conditions (44–46). Trace elements such as selenium, boron, and zinc can impact bone metabolism and normal development of the skeleton (47, 48). Trace elements in the body maintain a specific concentration constantly, variation in the level of trace elements may impair the function of the bone and joint system and increase the prevalence of OA (49). A cross-sectional study found significantly lower concentrations of manganese, copper and zinc in cancellous bone of osteoporotic patients than in non-osteoporotic patients (50). Iron homeostasis has a vital function in joint health, with several studies finding a link between excess iron and both OA and hemophilic arthropathy (51, 52). A cross-sectional investigation employing a multivariable logistic regression model indicated that magnesium intake has an inverse relationship with both radiographic knee OA and joint space narrowing (53). Additionally, serum magnesium levels were found to be inversely related to serum high-sensitivity C-reactive protein in patients with early radiographic knee OA (54). Additionally, a community-based study highlighted the relationship between selenium levels and OA prevalence. It revealed that individuals with lower serum selenium concentrations exhibited a higher prevalence of OA (55). Selenium is essential for redox homeostasis and selenophosphate synthetase 1 downregulation decreases the synthesis of selenoproteins resulting in exacerbation of OA (56). A recent finding revealed that selenium attenuates OA progression in human chondrocyte cell lines and rats by modulating Nrf2 and NF-κB Pathways to enhance antioxidant capacity and reduce inflammatory responses (57)(**Figure 2**). Boron can effectively repair cartilage damage and antioxidant (58). Certain trace elements such as magnesium, selenium, and boron require further research for the treatment or prevention of OA.

Different concentrations of several trace elements may bring different impacts on OA. The effects of trace elements on OA are multiple, and different types and doses of trace elements and their mechanisms of action need to be researched more deeply. The relationship between some metabolites and OA is listed in **Table 2**. Iron homeostasis is regulated by complex processes, which are important for the health of articular cartilage. A study has shown that excessive or deficient iron intake increases the risk of knee OA progression (59). Biochanin A can reduce intracellular iron concentration and rescue chondrocytes killed by iron to prevent iron overload associated with knee OA (52). Several studies have shown a positive association between cadmium and the risk of OA (60, 61), and yet a study has shown that low-dose cadmium may reduce joint inflammation (62). A Copper-incorporated bioactive glass-ceramics can heal cartilage Lesions and prevent the development of OA (63). Manganese and moderate concentrations of zinc may prevent OA progression, while zinc overload will damage articular cartilage (64).

**Table 2:** The relationship between metabolic disorders and osteoarthritis.

Authors	Year	Research object or gene	Metabolic process	Journal	Key findings
H. Li et al. (53)	2017	magnesium	Trace element metabolism	*Mod. Rheumatol.*	In patients with early radiographic knee OA, serum Mg was inversely correlated with serum hsCRP
D. Kang et al. (55)	2022	SEPHS1, selenium	Trace element metabolism	*Nat. Commun.*	SEPHS1 plays an important role in the regulation of selenium metabolism, and its downregulation can exacerbate OA by disrupting redox homeostasis
H.-L. Cheng et al. (56)	2024	selenium	Trace element metabolism	*Int. J. Mol. Sci.*	Selenium reduces OA by promoting the gene expression of glutathione synthesis-related enzymes through the Nrf2 pathway to enhance antioxidant capacity and down-regulating the NF-κB Pathway to reduce inflammation
M. Korkmaz et al. (57)	2019	Boron	Trace element metabolism	*Biol. Trace Elem. Res.*	Hyaluronic acid is superior to boron in repairing osteochondral defects, but boron has a better antioxidant capacity than hyaluronic acid
L. Wu. et al. (58)	2022	Iron	Trace element metabolism	*Nutrients*	High or low iron intake can exacerbate knee OA
L. Fang et al. (59)	2023	cadmium	Trace element metabolism	*Chemosphere*	Positive correlation between cadmium and OA
R. Lin et al. (62)	2019	Copper	Trace element metabolism	*Theranostics*	A copper-incorporated bioactive glass-ceramics was found to prevent OA by promoting cartilage regeneration and reducing joint inflammation.
M. Arra et al. (68)	2020	glycolysis-related genes	Glucose metabolism	*Nat. Commun.*	Chondrocytes that are exposed to inflammatory conditions display increased levels of glycolysis-related gene expression, indicating a shift in their cellular metabolism towards glycolysis
Z.Z. Li et al. (70)	2020	PKM2	Glycolytic pathway	*J. Cell. Biochem.*	Treatment of chondrocytes with IL-1β induces endoplasmic reticulum stress, which worsens joint inflammation and leads to apoptosis; however, the deletion of PKM2 reduces osteoarthritis by counteracting its effects via inhibition of the Rspo2/Wnt/β-catenin signaling pathway
C. Wang et al. (74)	2021	GLUT1	Glucose metabolism	*Bone Res.*	Ablation of GLUT1 leads to decreased expression of anabolic-related genes and increased expression of catabolic genes in chondrocytes, exacerbating OA
K. Li et al. (75)	2022	mice with type I diabetes, GLUT1	Glucose metabolism	*FASEB J.*	In the articular cartilage of type 1 diabetic mice subjected to medial meniscus destabilization surgery, GLUT1 expression is decreased, and the glycolysis rate is lowered, resulting in more severe cartilage damage in diabetic mice relative to their nondiabetic counterparts.
B. Liu et al. (78)	2024	inf-exo	Glycolytic pathway	*Adv. Sci.*	Inf-exo enhances M1 polarization and glycolysis in macrophages to induce synovitis and exacerbate OA
J. Yang et al. (79)	2024	MVPs,YAP1	Glycolytic pathway	*Adv. Sci.*	MVPs, a drug delivery system, can attenuate the progression of diabetic OA by inhibiting M1 macrophage infiltration and glycolysis via the YAP1/TXNIP signaling axis
D. Medina-Luna et al. (86)	2017	free fatty acids	Fatty acid metabolism	*Lipids Health Dis.*	Free fatty acid treatement of human chondrocytes leads to cellular oxidative stress and increased secretion of inflammatory factors, which may exacerbate OA
L. Tan et al. (88)	2021	Palmitate	Fatty acid metabolism	*Plos one*	Saturated fatty acid palmitate can promote chondrocyte apoptosis, endoplasmic reticulum stress and secretion of inflammatory factors to aggravate OA
Y. Xie et al. (91)	2019	Docosahexaenoic acid	Fatty acid metabolism	*Biomed. Pharmacother.*	In a rat model, Docosahexaenoic acid attenuates cartilage degeneration and inhibits blood vessel formation, which alleviates OA
T. Xiong et al. (92)	2024	n-3 polyunsaturated fatty acids	Fatty acid metabolism	*Int. Immunopharmacol.*	n-3 polyunsaturated fatty acids can attenuate the progression of obesity-associated OA by inhibiting the HMGB1-RAGE/TLR4 signaling pathway
Y. Chen et al. (93)	2018	GPR120,docosahexaenoic acid	Fatty acid metabolism	*Arthritis Res. Ther.*	Docosahexaenoic acid could alleviate OA by activating GPR120 to increase anti-inflammatory effects
X. Li et al. (95)	2024	Oleic,Linoleic acid	Fatty acid metabolism	*Biochim. Biophys. Acta, Mol. Basis Dis.*	Treatment of chondrocytes with oleic and linoleic acids reduces the expression of FOXO1 and SIRT1, thereby inhibiting chondrocyte autophagy, which aggravates OA
W.S. Choi et al. (99)	2019	CH25H,CYP7B1	Cholesterol metabolism	*Nature*	The CH25H - CYP7B1-RORα axis of cholesterol metabolism can regulate OA progression by modulating the expression of catabolism-related genes in chondrocytes
C. Cao et al. (101)	2022	LRP3	Cholesterol metabolism	*Nat. Commun.*	Knockdown of LRP3 (low-density lipoprotein receptor-related protein 3) can aggravate OA by up-regulating syndecan-4 through the Ras signaling pathway to regulate extracellular matrix metabolism in chondrocytes
R.Z. Hamza et al. (107)	2020	Lecithin	Phospholipid metabolism	*Molecules*	In a monoiodoacetic acid (MIA)-induced experimental OA (OA) model, lecithin reduces the secretion of inflammatory factors and the expression of catabolism-related genes to attenuate OA
G. Zhai et al. (108)	2019	Ratio of serum lysophosphatidylcholine (Lysopc) to phosphatidylcholine (PC)	Phospholipid metabolism	*Sci. Rep.*	The ratio of serum lysophosphatidylcholine (Lysopc) to phosphatidylcholine (PC) correlates with cartilage volume loss in OA
Y. Wei et al. (110)	2021	Phospholipase A2	Phospholipid metabolism	*Sci. Adv.*	Micellar nanoparticles loaded with phospholipase A2 inhibitors can attenuate cartilage degeneration and inflammatory response to alleviate OA
W. Zhang et al. (115)	2016	arginine	Amino acid metabolism	*Osteoarthr. Cartil.*	Arginine breaks down to produce ornithine and proline to relieve osteoarthritis
J. Li et al. (116)	2018	L-arginine	Amino acid metabolism	*Int. J. Biol. Macromol.*	Combination of l -arginine and allopurinol reduces expression of inflammatory factors in OA
G. Yang et al. (117)	2016	Beta-alanine	Amino acid metabolism	*Anal. Bioanal. Chem.*	Beta-alanine is associated with the sclerosis of subchondral bone.
J.R. Anderson et al. (118)	2020	Ex vivo equine cartilage explants	Amino acid metabolism	*J. Proteome Res.*	TNF-α and IL-1β treatment of Ex vivo equine cartilage explants identified lysine and isoleucine expression to be upregulated and alanine expression to be downregulated
Y. Kaneko et al. (119)	2019	N-acetyl cysteine	Amino acid metabolism	*Sci. Rep.*	N-acetyl cysteine reduces the expression of catabolism-related genes and apoptosis in rat chondrocytes, thereby attenuating OA
R. Chen et al. (120)	2018	Multiple amino acids	Amino acid metabolism	*J. Chromatogr. B:Anal. Technol. Biomed. Life Sci.*	Metabolomic analysis revealed altered expression of several amino acids in OA patients, with Alanine, γ-aminobutyric acid and 4-hydroxy-l-proline being important biomarkers for differentiating between diseased and normal populations, and the most significant metabolic effects of alanine, aspartic acid, glutamic acid, arginine and proline
O. Senol et al. (122)	2019	Branched chain amino acid(BCAA)	Amino acid metabolism	*Clin. Rheumatol.*	BCAA may be a potential biomarker for OA with elevated expression in OA.
Tootsi, K et al. (123)	2020	Serum levels of amino acids	Amino acid metabolism	*Metabolites*	Serum arginine levels positively correlate with imaging severity of OA and glycine levels negatively correlate with OA severity
Y. Wu et al. (124)	2023	ADMA	Amino acid metabolism	*Sci. Adv.*	ADMA blocks the deubiquitination of SOX9 by USP7 to induce chondrocyte degeneration and senescence, thereby aggravating OA
X. Wu et al. (127)	2023	U-13C	TCA cycle	*OSTEOARTHR CARTILAGE*	[U-13C] glucose labelling reveals that less glucose enters the tricarboxylic acid cycle in OA
S. Abdelrazig et al. (128)	2021	Metabolites in urine	TCA cycle	*Metabolomics*	Urinary metabolic profiles are altered in patients with OA, and these metabolic alterations are associated with disturbances in the TCA cycle and amino acid metabolism, among others
L. Liu et al. (130)	2023	α-ketoglutarate	TCA cycle	*Redox Biol.*	α-ketoglutarate attenuates OA by inhibiting the expression of catabolism-related genes, promoting the expression of anabolism-related genes, promoting mitochondrial autophagy, and decreasing oxidative stress
R. He et al. (131)	2024	α-ketoglutarate	TCA cycle	*Cell. Mol. Biol. Lett.*	α-ketoglutarate can alleviate OA by inhibiting the ETV4/SLC7A11/GPX4 signaling pathway

Summary of the relationship and molecular mechanisms between metabolic disorders and OA. SEPHS1, Selenophosphate synthetase 1; PKM2, pyruvate kinase 2; GLUT1, Glucose transporter type 1; HK2, hexokinase 2; inf-exo, inflammatory fibroblast-like synoviocyte-derived exosomes; MVPs, M1 macrophage membrane-camouflaged Verteporfin (Vt)-loaded PLGA nanoparticles; LRP3, low-density lipoprotein receptor-related protein 3; ADMA, asymmetric dimethylarginine.

## 5 Role of glycolytic pathway

Glucose metabolism is a complex biochemical process that encompasses several key pathways, including glycolysis, the pentose phosphate pathway, and the tricarboxylic acid (TCA) cycle (65). Glycolysis is fundamental for the breakdown of glucose to generate energy, and several enzymes are playing a critical role in glycolysis, such as hexokinase(HK), and pyruvate kinase (PKM) (66). Chondrocytes are constantly maintained in a low-oxygen environment due to the special structure of articular cartilage (67). In the hypoxic environment, the TCA cycle is inhibited, and glycolysis will become the main metabolic pathway, converting glucose into available energy (68). Research indicates that RNA sequencing of primary chondrocytes exposed to IL-1β for 24 hours, when compared to untreated chondrocytes, reveals a notable rise in the expression of glycolysis-related genes, including pyruvate kinase, lactate dehydrogenase, and HK2 (69). This finding implies that under inflammatory conditions, the metabolic pathway of chondrocytes shifts towards glycolysis. PKM2 serves as the primary regulatory enzyme that drives glycolysis, exhibiting higher expression levels in OA chondrocytes than in normal articular cartilage (70). Furthermore, the inhibition of PKM2 reduces endoplasmic reticulum stress-induced apoptosis and inflammatory damage in chondrocytes treated with IL-1β by antagonizing the Rspo2/Wnt/β-catenin signaling pathway (71). Attenuation of PKM2 is probably an effective therapy for OA. Dysregulation of glycolytic metabolism contributes to chondrocyte enlargement and the breakdown of the extracellular matrix, which accelerates the progression of OA (72, 73).

Glycolysis has a crucial role in OA and understanding its mechanism of function is crucial for the treatment of OA (74). Measuring the expression of genes associated with different glucose transporter proteins in chondrocytes, revealed that the expression level of GLUT1 was significantly elevated in rib chondrocytes and joint chondrocytes compared to other glucose transporter proteins (75). This suggests that the GLUT1-encoded glucose transporter protein plays a leading role in chondrocytes. The reduction in the expression levels of the anabolic genes ACAN and COL2A1 was significant, while the expression of the catabolic markers ADAMTS5 and MMP13 exhibited a notable increase following the ablation of GLUT1 in primary articular chondrocytes. This shift contributes to the advancement of OA and highlights the crucial role of GLUT1 in preserving the homeostatic balance of articular cartilage (75). In Type I diabetic mice, the expression of Glut1 and the rate of glycolysis were reduced in articular cartilage, and diabetic mice exhibited more severe cartilage destruction after DMM than non-diabetic mice (76). HKs catalyze the first step in glucose metabolism and HK2 is its major subtype (77). It has been found that treatment of human chondrocytes with TGF-β1, which maintains chondrocyte homeostasis, stimulates glycolysis by upregulating HK2 (78), indicating that HK2 could serve as a potential target for therapy in OA. Inflammatory fibroblast-like synoviocyte-derived exosomes promote the polarization of macrophages towards the M1 phenotype and increase glycolysis, exacerbating OA (79). Recently, researchers have created M1 macrophage membrane-camouflaged Vt-loaded PLGA nanoparticles, which may slow the advancement of diabetic OA by reducing infiltration of M1 macrophages and glycolysis through the YAP1/TXNIP signaling pathway (80). There is a significant relationship between glycolysis and OA, suggesting that developing inhibitors of glycolysis could offer innovative therapeutic strategies for preventing and treating OA.

## 6 Role of lipid

Lipids are bioactive molecules that are ubiquitous in the body and are the main structural and functional components of biological organisms, playing a key role in various biological processes (81), such as energy transfer, signal transmission, cell proliferation, and programmed cell death (82). Growing evidence emphasizes the significant involvement of lipids in the onset and progression of various diseases, including metabolic diseases, cardiovascular and renal diseases, cancer and liver diseases, and neurological disorders (83). The lipid accumulation product (LAP) can reflect the index of lipid over-accumulation in human body,A recent cross-sectional study utilizing the National Health and Nutrition Examination Survey for America found that the LAP index is significant for predicting and diagnosing OA (84). Lipid metabolism is probably involved in the pathogenesis of OA, playing a key role in cartilage growth, injury, and regeneration (85).

### 6.1 Fatty acid

Fatty acids (Fas) are available as a source of energy in conditions of nutrient deficiency, can be absorbed from food, and play a key role in several vital biological processes that are essential for maintaining cell function and tissue homeostasis (86). Human chondrocytes exposed to free Fas result in increased oxidative stress, elevated ROS levels, and increased secretion of cytokine IL-6 and chemokine IL-8, which can lead to chondrocyte damage (87). Palmitate is the major saturated FA in the human diet (88). Mice that were given a diet high in palmitate exhibited more significant cartilage damage, synovial hyperplasia, and heightened expression of ER stress and apoptotic markers (89). Additionally, there was an increase in serum pro-inflammatory markers, such as IL-6 and TNF-α, when compared to control groups. This indicates that ER stress induced by palmitate may contribute to cartilage damage and could represent a potential therapeutic target for OA (89). After IL-1β treated human and mouse chondrocytes, the expression of the receptor for medium-chain Fas (MCFA), GPR84, was found to increase, and the deletion of GPR84 resulted in increased expression of regulators of cartilage catabolism. Activation of GPR84 or the addition of MCFA may trigger the expression of genes associated with cartilage anabolism, thereby offering protection against the degeneration of cartilage and hindering the advancement of OA (90), and the beneficial effect of GPR84 on OA cartilage provides a direction for the treatment of OA. Omega-6 Fas and Omega-3 Fas are classified as unsaturated Fas, with established anti-inflammatory properties for Omega-3 Fas (91). Anterior cruciate ligament transection (ACLT) model is a commonly used animal model of OA. In research involving ACLT-operated rats treated with Omega-3, there was a noticeable reduction in the levels of MMP-13 and vascular endothelial growth factor when compared to the control group, indicating that omega-3 could alleviate cartilage degeneration and inhibit the formation of blood vessels, and protect cartilage in rats (92). A recent study found that n-3 polyunsaturated Fas (PUFA) could alleviate the progression of obesity-associated OA through modulation of the HMGB1-RAGE/TLR4 signaling pathway (93). Docosahexaenoic acid (DHA) is an unsaturated FA belonging to the Omega-3 family. DHA activates G-protein coupled receptor 120(GPR120) and exhibits anti-inflammatory effects in primary human chondrocytes cultured *in vitro*, suggesting that downregulation of GPR120 can interrupt metabolic homeostasis and that GPR120 levels can be considered as a diagnostic marker for OA (94). however, one study found that Omega-3 PUFAs were negatively associated with OA in adults aged 40 ~ 59 years, presenting the opposite results from previous studies (95). Linoleic acid is one of the omega-6 Fas, an essential FA for the body, oleic and linoleic acids can aggravate OA progression by downregulating the SIRT1/FOXO1 pathway (96), but a study has also found that omega-6 Fas can reduce the risk of OA (97), the emergence of such contradictory results indicates that different omega-6 Fas may have different effects for OA and that further research is needed to clarify the association between omega-6 Fas and OA.

### 6.2 Cholesterol metabolism

The accumulation of excess or deficient cholesterol in the body will lead to disease, and the relationship between high levels of cholesterol and atherosclerosis has been confirmed (98). Further understanding of cholesterol has revealed that abnormal cholesterol metabolism is linked to the onset of various diseases, including diabetes, chronic kidney disease, osteoporosis, Alzheimer’s disease, and OA (99). Cholesterol 25-hydroxylase (CH25H) plays a key role in cartilage cholesterol metabolism, and the CH25H-CYP7B1-RORα axis of cholesterol metabolism regulates OA via the upregulation of matrix-degrading enzymes exerting catabolic functions in chondrocytes (100). Additionally, MicroRNA-10A-3p reduces the production of pro-inflammatory factors induced by CH25H and reduces extracellular matrix (ECM) degradation, thereby improving cartilage degeneration. This effect is achieved through modulation of the CH25H-CYP7B1-RORα pathway in cholesterol metabolism (101). A recent study revealed that LRP3 (low-density lipoprotein receptor-related protein 3) expression was downregulated in degenerating human OA cartilage and C57BL/6 mice models, that LRP3 gene deletion aggravated cartilage degeneration in mouse models, and that downregulation of LPR3 activated the Ras/Raf/MEK/ERK signaling pathway to upregulate SDC4 expression to induce cartilage ECM degeneration and promote OA progression, indicating that the cholesterol-LRP3-SDC4 axis is involved in regulating chondrocyte homeostasis and may provide options for the treatment of OA (102). The association between elevated serum cholesterol levels and OA and the role of cholesterol in the pathogenesis of OA requires further research, which will probably provide more therapeutic avenues for the treatment of OA.

### 6.3 Phospholipid metabolism

Phospholipids are critical components of cell membranes, and the liver and kidneys are the major production areas for phospholipids, which play an important role in signal transduction (103). According to the glycerol backbone, phospholipids can be classified as glycerophospholipids and sphingolipids (104). Glycerophospholipids consist of phosphatidylcholine (PC), phosphatidylethanolamine (PE), phosphatidylserine, phosphatidylinositol, and phosphatidic acid (103). Among these, glycerophospholipid is recognized as the most prevalent phospholipid within the organism (105). Using global metabolomic profiling of synovial fluid found phospholipids as potential biomarkers of OA and contribute to joint lubrication (106, 107). Monoiodoacetate (MIA) induced experimental OA model, and the addition of lecithin decreased the levels of IL-6 and CRP and significantly reduced the production of ROS compared to the positive OA control treated group, which alleviates the joint damage caused by MIA-induced experimental OA model (108). A clinical study showed that the serum ratio of lysophosphatidylcholine(LysoPC) to PC is closely related to cartilage volume loss over time in symptomatic knee OA patients, so the serum level ratio of lysoPC to PC might serve as a potential biomarker for the progression of OA (109). Phospholipase A2 is an enzyme that catalyzes the hydrolysis of glycerophospholipids, releasing arachidonic acid and lysophospholipids. Arachidonic acid is crucial for the modulation of inflammation progression (110). A study revealed that Phospholipase A2 levels were markedly elevated in OA cartilage in comparison to normal cartilage, and Phospholipase A2 inhibitor-loaded micellar nanoparticles could improve the cartilage degeneration and inflammatory response caused by OA in a surgery-induced mouse OA model, providing a new therapeutic strategy for the treatment of OA (111).

## 7 Role of amino acid metabolism

AAs are not only components of proteins and peptides, but also essential bioactive molecules in the body, playing a key role in regulating various biological processes, including signaling pathways, metabolic regulation, energy homeostasis, and as potential biomarkers of various diseases (112, 113). AAs are categorized into two groups: essential and non-essential. AAs that cannot be synthesized from scratch and must be obtained from food are essential AAs, including lysine, isoleucine, phenylalanine, tryptophan, valine, methionine, leucine, threonine, and histidine (114, 115). Branched chain AAs consist of leucine, isoleucine, and valine, and they represent the most abundant essential AAs found in the body (112). Through studies in recent years, AAs metabolism disorders were found to possibly be related to the pathogenesis of OA, and AAs expressed in OA with altered levels could serve as potential biomarkers of the disease, including arginine, branched chain AA, and alanine (107). Zhang, S et al. revealed that active arginine catabolism in OA generates ornithine and proline to repair damaged cartilage to alleviate osteoarthritis (116). Combination of L-arginine and allopurinol significantly reduces the expression of inflammatory factors in OA (117). Beta-alanine is associated with subchondral osteosclerosis in OA (118). Consequently, it is necessary to study the changes in AAs metabolism in OA and its mechanism of function.

A study found that treated equine cartilage explants with TNF-α/IL-1β resulted in increased lysine levels and decreased alanine expression (119). N-acetyl cysteine (NAC) is an antioxidant, Oral administration of NAC could significantly inhibit the down-regulation of MMP13 and type II collagen expression in a rat OA model and could inhibit chondrocyte apoptosis, which could effectively prevent the occurrence and development of OA (120). Through metabolomic analysis, researchers identified increased levels of 10 metabolites (leucine, arginine, valine, isoleucine, tryptophan, alanine, lysine, creatine, tyrosine, and 4-hydroxy-L-proline) and a reduction in the concentration of metabolites (glutamine, phenylalanine, serine, proline, γ-aminobutyric acid, creatinine, dimethylglycine, taurine, asparagine, aminobutyric acid, acetyl-carnitine, and citrulline) in patients with OA. Among these, alanine, γ-aminobutyric acid, and 4-hydroxy-L-proline may serve as potential biomarkers for OA (121). Branched chain AA and its metabolites are critical in the regulation of skeletal muscle function (122), Senol et al. suggest that branched chain AA is more highly expressed in OA and could be a potential biomarker for OA (123). Compared to controls, elevated arginine levels were found in the serum of OA patients and showed a positive correlation with severe imaging severity in OA, and decreased glycine levels in the severe OA patients (124). A recent study found that asymmetric dimethylarginine binds to USP7 and SOX9, resulting in the degradation of SOX9, which promotes chondrocyte degeneration to promote the progression of OA (125). In conclusion, from the perspective of metabolism, metabolic disorders of AA are involved in the pathogenesis of OA, and an in-depth understanding of the relationship between them can provide some assistance in the diagnosis and treatment of OA.

## 8 Relationship between OA and TCA cycle

In the pathogenesis of OA, metabolic pathways are linked to varying degrees and don’t function independently. The TCA cycle is a common oxidation pathway for sugars, lipids, and proteins. It is the main pathway providing energy sources to the body dominating metabolism (126), and playing a role in chromatin modifications, and DNA methylation (127). TCA cycle might be involved in the pathogenesis of OA. In OA patients, [U-13C] glucose isotope labeling revealed decreases in glucose-derived carbon entering the TCA cycle, as well as significant decreases in mitochondrial respiration rate and increases in basal extracellular acidification rate in chondrocytes (128). In the urine of patients with OA, increased concentrations of acetylphosphate, fumarate, and s-lac-toylglutathione indicate a significant upregulation of the pyruvate pathway and TCA cycle (129). Acetyl-CoA derived from glucose, Fas, and AAs is a substrate of the TCA cycle, and a study showed that acetyl-CoA is crucial for regulating the maturation of chondrocytes (130). α-ketoglutaric acid acts as a vital metabolic intermediate in the TCA cycle and serves as a precursor for glutamate and glutamine; its levels in serum tend to decline with advancing age. A study revealed that supplementation with α-ketoglutarate can alleviate OA (131). Recently, an *in vivo* study found that α-Ketoglutarate alleviated OA by inhibiting ferroptosis through the ETV4/SLC7A11/GPX4 signaling pathway (132).

## 9 Conclusions

In conclusion, the pathogenesis of OA is complex, and recent studies have revealed that metabolic disorders are probably involved in the pathogenesis of OA and promote OA progression. The association between the metabolic syndrome and its components and OA needs to be researched more comprehensively which needs to consider the impact of BMI. Several trace elements such as magnesium, boron, and selenium may prevent the progression of OA, with different levels of trace elements bringing different effects. The increased expression of glycolysis-related genes in OA and the shift to glycolysis in chondrocyte metabolism both contribute to the progression of OA. MCFA receptor GPR84 could induce the expression of cartilage anabolic-related genes to prevent the progression of OA, providing a new strategy for the treatment of OA. Recent studies revealed the protective effects of Omega-3 FAs and Phospholipase A2 inhibitor-loaded micellar nanoparticles on rat cartilage. Arginine, alanine, BCAA, and 4-hydroxy-L-proline were found to have elevated expression in OA and could serve as potential biomarkers of OA, and supplementation with BCAA may alleviate the progression of OA. α-ketoglutarate plays an important role in the diagnosis and treatment of OA and may have potential clinical value in the future. In conclusion, the improvement of metabolic disorders in patients with OA is beneficial for the treatment of the disease.

The review focuses on the role of various metabolites in OA and their relationship to OA. However, the mechanisms of action of metabolic disorders in OA require more comprehensive studies and larger studies in OA patients, combining proteomics, metabolomics, and Lipidomics to get an in-depth understanding of the alteration of metabolic signaling in OA and identify potential biomarkers and therapeutic targets for OA. Recently research on the relationship between lipids and OA has gradually increased and presented different results, which requires more specific studies to elaborate on the relationship between the two. The current early diagnosis and treatment of OA is not effective, and future studies should focus more on the link between metabolism syndrome and OA, and possibly identify new biomarkers and therapeutic targets for OA.
